# REPORT ON THE INTERIM MEETING OF THE NATIONAL CONFERENCE ON WEIGHTSAND MEASURES JANUARY 12–16, 1987

**DOI:** 10.6028/jres.092.027

**Published:** 1987-08-01

**Authors:** Carroll S. Brickenkamp

**Affiliations:** Office of Weights and Measures, Office of Product Standards Policy, National Bureau of Standards, Gaithersburg, MD 20899

The National Conference on Weights and Measures (NCWM) is a standards-writing organization that dates its establishment back to January 16, 1905, when the Director of the National Bureau of Standards hosted the first meeting of nine State representatives “in order to bring about uniformity in the state laws referring to weights and measures, and also to effect a close cooperation between the state inspection services and the National Bureau of Standards….” Today, all the NCWM technical committees and task forces meet at NBS in January of each year at what is called the “NCWM Interim Meeting” to study issues and make recommendations on weights and measures concerns, particularly the regulation of the commercial marketplace–scales, liquid meters, and prepackaged commodities. The active membership of the NCWM meet annually in July to vote on the Committees’ recommendations. The Interim Meeting of the 72d NCWM (gaps in Annual Meetings occurred during both world wars) was held January 12–16, 1987. A summary of the most significant work of each major committee follows.

The Executive Committee manages the NCWM, sets policy, and sits as the Board of Governors of the National Type Evaluation Program (NTEP). The NTEP is a program of cooperation between NBS, NCWM, States, and the private sector for determining conformance of measuring devices with codes and other legal design criteria. A survey indicates that the program is accepted in all but three States in the United States. This year, the Executive Committee is revising the Policy and Procedures under which NTEP operates. Of special significance was the completion of plans for the evaluation of load cells used in commercial weighing devices. The NBS Automated Production Technology Division will provide the technical oversight for this activity. In addition, the Executive Committee is recommending new policies for interaction with the International Organization for Legal Metrology (OIML) in order to communicate the work of the OIML to a broader audience within the U.S.

The Committee on Laws and Regulations develops and interprets standards that are recommended for adoption by States as laws and regulations. This year, the Committee is proposing adoption by the NCWM of a Uniform Motor Fuel Law. This law incorporates ASTM standards on gasoline, diesel fuel, and gasoline-alcohol blends. In addition, the Committee is recommending test methods for packaged commodities subject to moisture loss.

The Committee on Specifications and Tolerances develops standards that are adopted by States as device code requirements and test methods. At the 1987 Interim Meeting, the Committee determined how the NCWM will set standards and test procedures to deal with the problem of gasoline vaporization during tests of “loading-rack” (wholesale gasoline delivery) meters.

The Committee on Education promotes the education and training of weights and measures officials for an increasingly technologically-sophisticated marketplace. It has managed the development of a series of training “modules” under a grant from the NBS. The Committee has proposed minimum qualifications this year for selecting instructors to deliver the training using the modules.

The Committee on Liaison represents the NCWM to the Federal Government and other organizations. At the 1987 Interim Meeting, the Committee agreed to petition the Department of Treasury, Bureau of Alcohol, Tobacco, and Firearms to modify their regu1ations for malt beverages to conform to Federal and State labeling requirements covering other types of packaged goods.

The 72d Annual Meeting of the NCWM will be held in Little Rock, Arkansas Ju1y 19–24, 1987. Approximately 400 representatives from government and industry are expected to attend. The NBS will publish the proceedings of the meeting as a special publication, and publish updates of the weights and measures handbooks, Handbook 44 “Specifications, Tolerances, and Other Technical Requirements for Weighing and Measuring Devices,” Handbook 130 “Uniform Laws and Regu1ations,” and Handbook 133 “Checking the Net Contents of Packaged Goods” as a resu1t of voting at the Annual Meeting.

For further information call or write Albert D. Tholen, Office of Weights and Measures, Office of Product Standards Policy, National Bureau of Standards, Gaithersburg, MD 20899.

